# Applications of Omics Technology for Livestock Selection and Improvement

**DOI:** 10.3389/fgene.2022.774113

**Published:** 2022-06-02

**Authors:** Dibyendu Chakraborty, Neelesh Sharma, Savleen Kour, Simrinder Singh Sodhi, Mukesh Kumar Gupta, Sung Jin Lee, Young Ok Son

**Affiliations:** ^1^ Division of Animal Genetics and Breeding, Faculty of Veterinary Sciences and Animal Husbandry, Sher-e-Kashmir University of Agricultural Sciences and Technology of Jammu, Ranbir Singh Pura, India; ^2^ Division of Veterinary Medicine, Faculty of Veterinary Sciences and Animal Husbandry, Sher-e-Kashmir University of Agricultural Sciences and Technology of Jammu, Ranbir Singh Pura, India; ^3^ Department of Animal Biotechnology, College of Animal Biotechnology, Guru Angad Dev Veterinary and Animal Sciences University, Ludhiana, India; ^4^ Department of Biotechnology and Medical Engineering, National Institute of Technology, Rourkela, India; ^5^ Department of Animal Biotechnology, College of Animal Life Sciences, Kangwon National University, Chuncheon-si, South Korea; ^6^ Department of Animal Biotechnology, Faculty of Biotechnology, College of Applied Life Sciences and Interdisciplinary Graduate Program in Advanced Convergence Technology and Science, Jeju National University, Jeju, South Korea

**Keywords:** omics, selection, animal improvement, phenomics, data analysis

## Abstract

Conventional animal selection and breeding methods were based on the phenotypic performance of the animals. These methods have limitations, particularly for sex-limited traits and traits expressed later in the life cycle (e.g., carcass traits). Consequently, the genetic gain has been slow with high generation intervals. With the advent of high-throughput *omics* techniques and the availability of *multi-omics* technologies and sophisticated analytic packages, several promising tools and methods have been developed to estimate the actual genetic potential of the animals. It has now become possible to collect and access large and complex datasets comprising different genomics, transcriptomics, proteomics, metabolomics, and phonemics data as well as animal-level data (such as longevity, behavior, adaptation, etc.,), which provides new opportunities to better understand the mechanisms regulating animals’ actual performance. The cost of *omics* technology and expertise of several fields like biology, bioinformatics, statistics, and computational biology make these technology impediments to its use in some cases. The population size and accurate phenotypic data recordings are other significant constraints for appropriate selection and breeding strategies. Nevertheless, *omics* technologies can estimate more accurate breeding values (BVs) and increase the genetic gain by assisting the section of genetically superior, disease-free animals at an early stage of life for enhancing animal productivity and profitability. This manuscript provides an overview of various omics technologies and their limitations for animal genetic selection and breeding decisions.

## Introduction

Genetic selection and breeding are crucial tools for livestock improvement. They have resulted in genetically superior and disease-free animals with improved production and efficiency in various livestock species (Rexroad et al., 2019; Erasmus and van Marle-Köster 2021). In earlier days, the genetic selection of animals for breeding was primarily based on their phenotypic characteristics, such as production traits and breeding value (BV) estimation. Later, other economic traits, including reproduction and longevity traits, animal health, stress tolerance, disease resistance, animal welfare traits, etc., also became vital components of genetic improvement programs (Brito et al., 2020; Brito et al., 2021). Selective breeding of genetically superior animals ensured rapid genetic progress of production efficiency traits to the next generation. Many breeding techniques have thus, evolved to accrue the desired trait in genetically selected animals to meet the market demand for production and animal welfare (Plieschke et al., 2016).

Several selection indices have been developed for the genetic selection of animals for breeding. However, no single trait is ideal for these selection indices in all populations (Cole et al., 2021). Further, while animals for selective breeding can be identified based on phenotypic recordings, traits that are sex-limited, expressed at a later stage of life, difficult to measure, or have low heritability pose difficulties (Calus et al., 2013). The use of complex statistical models, advanced analytic tools, and new molecular methods may divulge newer traits and help identify animals for efficient genetic selection and breeding with greater accuracy (Stock et al., 2020).

The past three decades have seen tremendous advancements in molecular genetics that have provided a better genetic understanding of quantitative economic traits (Dekkers and Hospital 2002). A number of genes and gene combinations have been found to directly correlate with animal performance and production efficiency (Rexroad et al., 2019; Ruan et al., 2021). Many quantitative trait loci (QTL)—gene loci responsible for trait diversity—have been identified for various production and reproductive traits and used for selection and breeding decisions (Zhang et al., 2021; Al-Sharif et al., 2022). Several genetic markers have also been discovered for use in marker-assisted selection (MAS) of breeding stock (Ma et al., 2021; Raza et al., 2021). More recently, with advancements in high throughput *omics* technologies, genome selection is becoming widely accepted for the selection of animals for breeding (Tan et al., 2017; Yang et al., 2020). The application of *omics* tools in livestock improvement may provide a more accurate technology for animal selection and breeding and therefore has become a hot spot of research (Pedrosa et al., 2021; Ruan et al., 2021). This manuscript provides an overview of various *omics* tools and technologies for their application in livestock selection and improvement programs.

## Omics Technology

**FIGURE 1:**
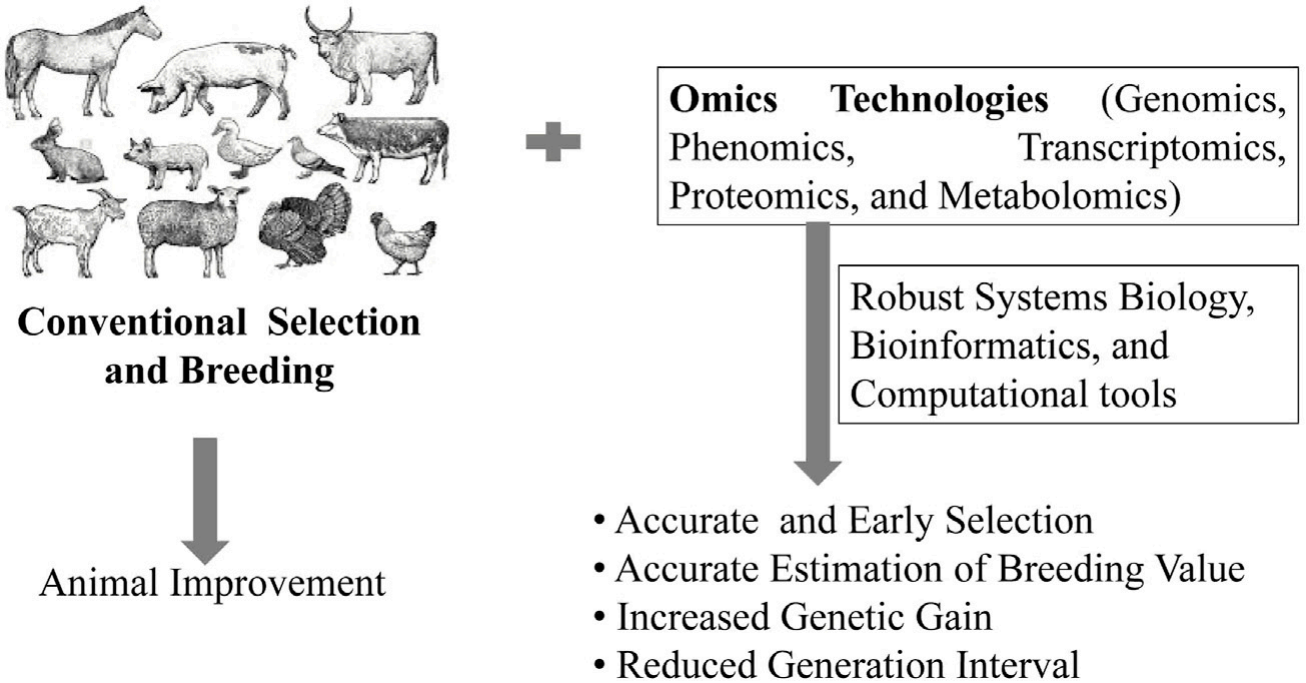
Impact of omics technology in animal improvement.

*Omics* technologies such as genomics, metagenomics, metabolomics, proteomics, transcriptomics, epigenomics, translatomics, etc., can allow rapid and effective detection of subtle phenotypic changes, dietary responses, and innate phenotypic propensities in animals (Mu et al., 2022; Wang et al., 2022). The utilization of *omics* tools in animal selection and breeding programs is thus, expected to provide an accurate estimation of BV for early selection, reduce generation interval and increase the rate of genetic gain (Figure 1). The word ‘*omics*’ originates from the suffix ‘*-ome’*, derived from a Greek word that means “whole”, “all” or “complete”. The suffix “*-omics”* is frequently used to refer to a field of study in life sciences that emphasizes large-scale high throughput data/information to understand life summed up in “omes” (Yadav, 2007). Several *omics* tools have been developed in the last two decades to collect and analyze high-throughput data on proteins (proteomics), mRNA transcripts (transcriptomics), gene sequences (genomics), microbial diversity (metagenomics), epigenetic regulation of gene expression (epigenomics), metabolic profile (metabolomics), lipid profile (lipidomics), etc., of a particular cell, tissue, organ or whole organism at a specific time point. The time (temporal) and space (spatial) level information from *omics* data can be integrated through robust bioinformatics and computational tools to the systems biology level (Odom et al., 2021; Velten et al., 2022). Network modeling of *omics* data can be used to study the mechanism, relationship, interaction, and function of cells, tissues, organs, and the whole organism at a molecular level in an unbiased manner (Aardema and MacGregor, 2003). More recently, *multi-omics* has emerged as high-dimensional biology (HBD) for simultaneous study of genetic variations in biological systems at the genes, transcripts, proteins, and metabolites level (Romero et al., 2006; Krassowski et al., 2020).

In the last two decades, a few landmark technological revolutions took place in *omics* technologies that revolutionized their application in genetic selection for animal breeding. In 2007, the first “high density” panel of bovine genetic markers was released commercially with a set of 54,001 single nucleotide polymorphisms (SNPs). Using such high-density SNP chips, genome-wide association studies (GWAS) demonstrated the link between SNPs and QTLs, such as coat color and presence or absence of horns (Matukumalli et al., 2009). In other studies, high-density SNP chips were shown to be useful in the genetic characterization of pig breeds for preserving their genomic variability (Muñoz et al., 2019). Whole-genome sequencing (WGS) by massively parallel sequencing was yet another breakthrough for detecting molecular signatures for the selection and breeding of animals (Elsik et al., 2009; Bovo et al., 2020). The Bovine Genome Sequencing and Assembly project (Elsik et al., 2009; The Bovine Genome Sequencing and Analysis Consortium et al., 2009; The Bovine HapMap Consortium, 2009) provided a landscape of genome sequence that subsequently led to a paradigm shift in QTL- and candidate gene-based approaches for genetic selection.

Molecular databases of NCBI (United States), EMBL (Europe), and DDBJ (Japan) provide vast information on nucleotide and protein sequences. These databases have been utilized in *omics* technology for understanding the genomic variability and molecular and physiological basis of economic traits (Wu et al., 2018; Ng et al., 2021). Unfortunately, however, very scant information is available on the precise regulatory networks through which these genes and proteins determine the phenotypic expression of economic traits. Further, a significant unexplained source of variation among phenotypes of various economic traits remains a matter of concern in livestock. Newer machine learning (ML) tools have been developed recently that can be exploited to analyze high throughput *omics* data, available in databases, for a greater understanding of gene regulatory networks (GRNs) and identification of functional genes by a systems biology approach (Guttula et al., 2020; Guttula et al., 2021; Ng et al., 2021).

*Omics* technologies can help identify functional SNPs and their prioritizing to increase the accuracy of genetic selection (Chang et al., 2019). They can also be used for selecting animals resistant to production diseases such as mastitis and thereby enhance their productivity (Russell et al., 2012; Bhattarai et al., 2017; Jaiswal et al., 2021). Further, population-level *omics* (e.g., population genomics) hold tremendous potential for classifying individuals based on allelic diversity and identifying genetically-related individuals (Lippert et al., 2017). Such strategies can help calculate homozygosity and inbreeding coefficients (Ghoreishifar et al., 2020; Sumreddee et al., 2021) for designing appropriate breeding programs to maintain genetic diversity and avoid inbreeding depression (Alemu et al., 2021; Bu et al., 2021). However, while many WGS databases and consortiums have been formed in humans (Zhang et al., 2018) (GenomeAsia 100K Consortium, 2019), no high-resolution database of population-level genetic variants is available for animals.

## Genomics

The genome is defined as the complete set of genetic material present in an organism. The term “genomics” was coined in 1986 by scientists who were naming a new journal (Kuska, 1998), and thus, the era of *omics* began. The major developments in genomics are the discovery of the genes and genetic codes, polymerase chain reaction (PCR), Genome sequencing by Sanger sequencing or Next Generation Sequencing (NGS), and genome editing tools such as Transcription activator-like effector nuclease (TALEN), Zinc-finer nuclease (ZNF), an Clustered regularly interspaced short palindromic repeats (CRISPR)/CRISPR-associated protein 9 (CRISPR/Cas9) technologies. The field of genomics started gaining popularity after the invention of PCR in the year 1985. Thereafter, MAS- and candidate gene-based approaches for selecting genetically superior animals became very popular and were found to be better than conventional phenotype-based selection and breeding (Williams 2005). Gene map construction was also used for genome sequencing. The gene map construction was initially based on the segregation of enzyme markers across panels of hybrid cell lines. However, with advancements in recombinant DNA (rDNA) technology, denser physical and genetic maps formed an important framework for genome sequencing (Riggs and Gill, 2009). During the early 2000s, most livestock genome sequencing was based on linkage maps using single markers and quantifying one or a few genes by real-time quantitative PCR (qPCR). Elsik and associates, in 2009, published the first bovine genome assembly (Elsik et al., 2009). Since then, rapid progress has been made in developing and using several whole genome-*omics* tools that have accelerated cattle genetics research (Reverter et al., 2013; Snelling et al., 2013).

The concept of “Genetical Genomics”, which integrates structural and functional genomic data, has evolved with the development of microarray technology for gene expression analysis, which divulged marker genotypes across whole genome. The field of Genetical Genomics has expanded with the availability of high throughput tools for genomic analysis such as high-density (HD) genotyping-chips (Illumina, San Diego, CA), WGS, genotyping by sequencing, and RNA-sequencing (RNAseq) to measure the gene expression in the entire transcriptome (Wickramasinghe et al., 2014). Several SNP chips of 60 K for pig and chicken, 50 K for sheep, and 77 K for cattle have also been developed (Suravajhala et al., 2016). The GWAS studies have become very popular among different livestock species focusing on production and health traits (Shamra et al., 2015). For example, GWAS on female reproduction traits in tropically adapted beef cattle (Hawken et al., 2012), feed efficiency traits in pigs (Do et al., 2013 and 2014), body weight in broilers (Wang et al., 2014), and obesity and metabolic diseases using the pig as a model (Kogelman et al., 2014) have been conducted. The genomic selection is particulary advantagious as it can be used for selecting animals for breeding at an early stage of life without having reference to their own breeding or production records. Studies have shown that genomic selection improved the genetic gain as much as 60–120% in dairy cattle by decreasing genetic interval by 2 years, although the extent of added genetic gain was lower in other livestock species (Table 1). The term “systems genetics” or “systems genomics” was also proposed by Kadarmideen (2014). This branch has a wide range of approaches ranging from relating the individual’s *omics* levels data to their functional annotation and analysis of signaling pathways by integrating different multi-omic levels data to phenotypes.

**TABLE 1:** Impact of genomic selection^a^.

Animals	Added genetic Gain	References
Dairy cattle	60–120%	Pryce and Daetwyler, (2011)
Beef cattle	15–44%	Pimentel and Konig, (2012)
Dairy goat	26.2%	Shumbusho et al. (2013)
Dairy sheep	51.7%	Shumbusho et al. (2013)
Meat sheep	17.9%	Shumbusho et al. (2013)
Pig	23–91%	Lillehammer et al. (2011)
Layers	60%	Sitzenstock et al. (2013)
Broilers	20%	Dekkers et al. (2009)
Dairy Bulls	30–71%	Doublet et al. (2019)

a Source: Modified from Ibisham et al., 2017.

## Transcriptomics

Transcriptomic methods can be used to compare a biological response to different conditions or treatments or to assess physiological responses to external stimuli (Brannan et al., 2014). Whole transcriptome sequencing is the most widely used method for studying RNA functions, exploring and analyzing the gene structure and function, and revealing intrinsic links between gene expression and life phenomena (Shi and Zhang, 2019). To date, extensive research has been carried out in different livestock species using high-throughput RNAseq technology that has replaced the earlier used Maxam and Gilbert chemical degradation sequencing method. The NGS technologies generate sequence data by producing millions of short DNA fragments in parallel. The template is broken into many smaller fragments by sheering, which are then ligated to adapters to create cDNA libraries by the bridge (e.g., Illumina sequencing) or emulsion (e.g., pyrosequencing) PCR. The clones of cDNA fragments of each library are then sequenced to obtain short *reads*; the length and number of the reads vary with the specific technology but generally range between 30 and 300 bases, which is shorter than those obtained from Sanger sequencing (Ghaffari et al., 2013). NGS has led to the characterization and quantification of many omics, including genomics (DNA sequencing), transcriptomics (RNA and cDNA sequencing), and epigenomics (ChiP-seq and DNA methylation analysis). More recently, third-generation sequencing methods involving single-molecule real-time (SMRT) sequencing have emerged (Sahoo et al., 2021a; Sahoo et al., 2021b). These newer SMRT sequencing methods do not require PCR amplification of templates and hence are devoid of PCR biases. Moreover, the SMRT sequencing approaches generally produce longer reads for better genome assembly and identification of indels (Athanasopoulou et al., 2021). However, SMRT methods such as Nanopore^TM^ and PacBio^TM^ sequencing also offer versatility in terms of rapid time and the transportability of the equipment. Newer techniques such as tunneling currents DNA sequencing, sequencing with mass spectrometry, microscopy-based sequencing, etc., are under development.

The RNAseq has made a revolutionary impact on transcriptome analysis (Mortazavi et al., 2008). RNAseq has major advantages such as a large dynamic range and sensitivity, precise, unbiased quantification of transcripts, and comprehensive coverage of all expressed sequences in a given tissue sample. The direct RNAseq is vital for functional studies to capture the dynamic RNA population under different environmental conditions (Athanasopoulou et al., 2021). It has revolutionized gene annotation, which was hitherto very difficult with genome sequencing. The RNAseq also finds application in analyzing molecular features such as alternate isoforms, splice variants, fusion transcripts, RNA editing, etc. (Li and Wang 2021). Combination of genome sequencing with RNAseq can be utilized to interpret mutations on regulatory regions of genes, which do not produce an obvious effect on the protein sequence (Cohen et al., 2020).

**FIGURE 2:**
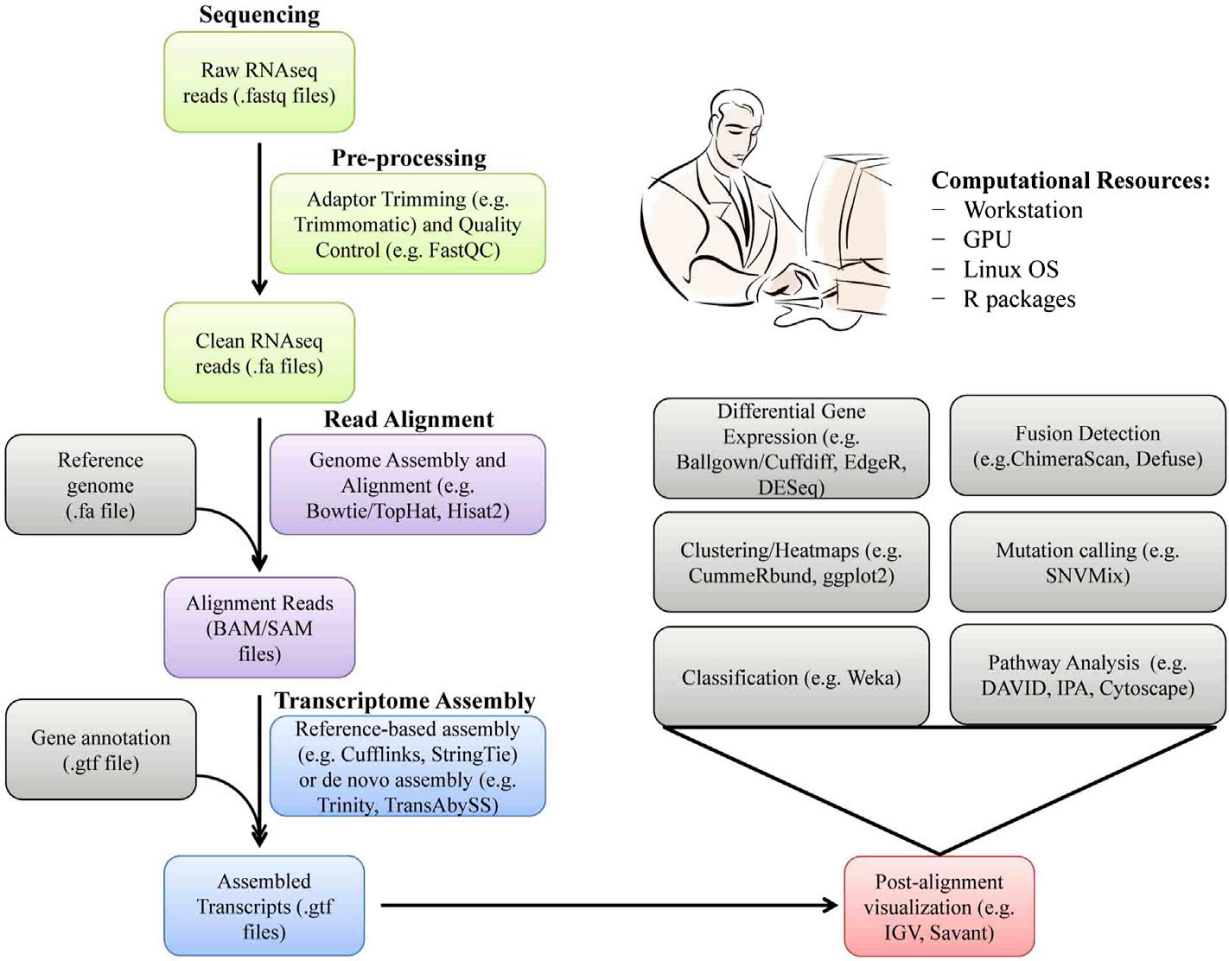
A software pipeline and computational resources used for analysis of RNAseq data. Each type of RNAseq has distinct requirements and challenges but there is a common workflow/pipeline.

Today, very accurate and efficient sequencing platforms are available, which can distinguish closely related transcripts from each other (Marguerat and Braga-Neto, 2015). Therefore, RNAseq has become very popular for the identification and quantification of splice variants, fused transcripts, and mutants. In RNAseq technology, messenger RNAs are first randomly fragmented into small pieces by sheering and converted to a library of complementary DNA (cDNA) fragments. These cDNA fragments are then amplified and sequenced in parallel and mapped to a given region of the target genome. PCR-free cDNA sequencing and direct RNAseq without first-strand cDNA synthesis have also become possible with SMRT technology such as Nanopore^TM^ sequencing. In expression quantification, a count, which is determined by the number of reads mapping to each gene (FPKM or TPM; fragments per kilobase of transcripts per million mapped reads or transcripts per million), is a discrete measure of the corresponding gene expression level (Ghaffari*et al.*, 2013, Li et al., 2012) (Figure 2). The differentially expressed genes (DEGs) between two samples can be obtained by transcript compilation with gene annotation file followed by gene identification and differential expression analysis based on FPKM or TPM values (Alessandrì et al., 2019). Functional analysis of DEGs by bioinformatics tools revealed that the immune and inflammatory responses were the most impacted pathways between purebred and crossbred cattle populations (Moridi et al., 2019). Such type of RNAseq-based transcriptomic studies on animals of high- and low-genetic merit may be helpful for the selection and breeding of elite animals in the future to enhance health, productivity, and profitability.

Canovas et al. (2014) integrated the RNAseq data with GWAS and bovine transcriptional factors in multiple tissues from pre- and post-pubertal cattle and constructed co-expression GRNs, which revealed genes and their complex interactions during puberty in cattle. Therefore, early selection of individuals based on *multi-omics* data from early sexual maturity may help increase the genetic gain by reducing generation intervals. In another study, the resistance or susceptibility of Creole goats to gastrointestinal nematodes was studied by RNAseq (Aboshady et al., 2021). Aboshady et al. (2021) reported that the T-cell receptor signaling pathway was one of the top significant pathways that distinguish the resistant from the susceptible genotype, with 78% of the genes involved in this pathway showing genomic variants in Creole goats. This shows another important example of applying *omics* for selecting disease-resistant animals.

## Proteomics

Wilkins and Williams first described proteomics in the mid-1990s (Speicher, 2004). Proteomics allows analysis of all proteins, including their isoforms, in a particular cell, tissue, or organ at a specific time in a single experiment. Advance proteomic tools can also provide information on various protein isoforms, their quantification, and protein-protein interaction. However, the application of proteomics in livestock research has been limited in the past due to its high cost and lack of optimized protocols for various cell types in different species (Baykalir et al., 2018). Nevertheless, with advancements in new analytical methods and computational tools for the analysis of proteomic data, reports on proteomics studies in animal science are increasing for understanding the animal health status and production and reproduction efficiency (Zhao et al., 2021; Kaya et al., 2022; Ye et al., 2022).

**FIGURE 3:**
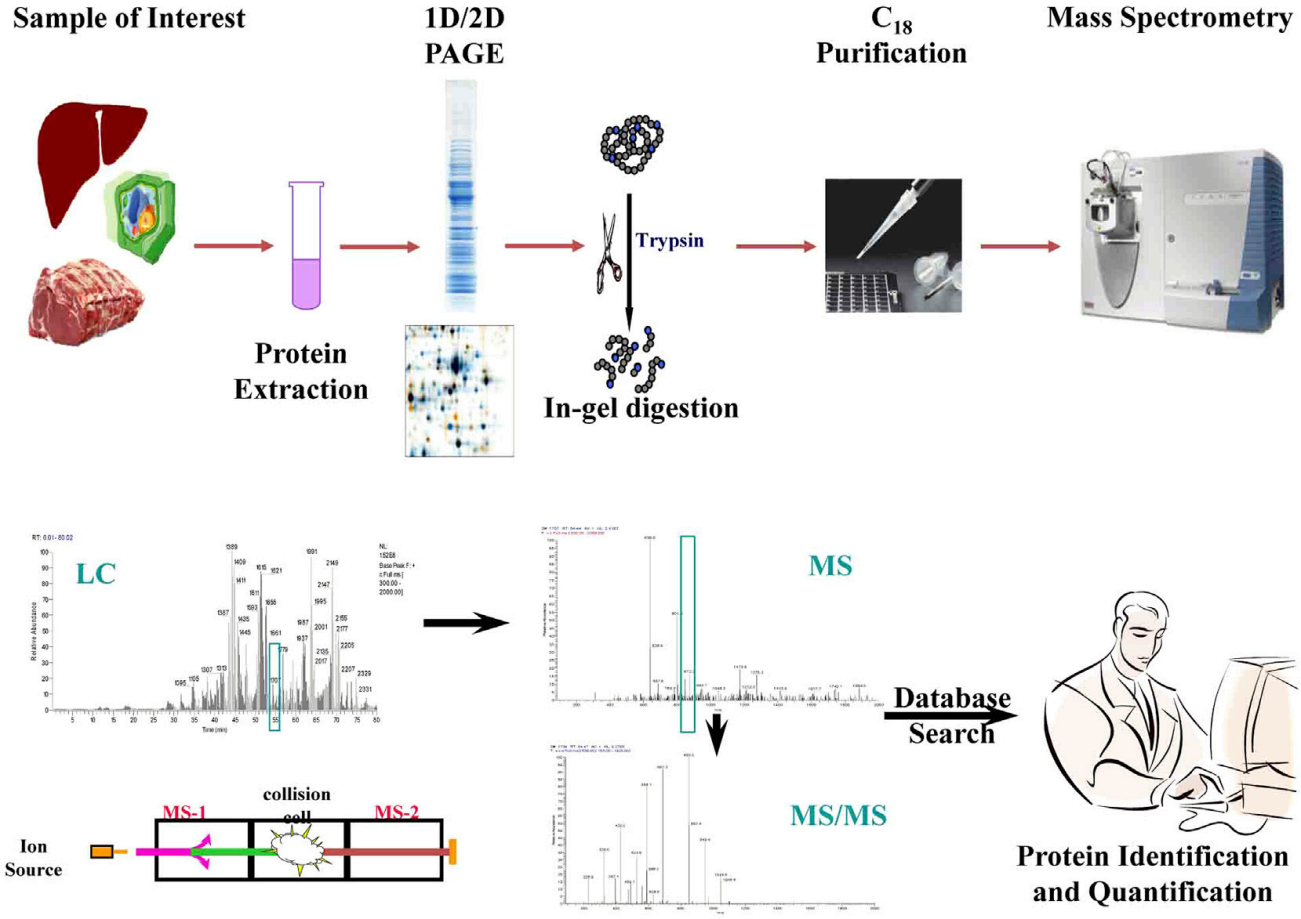
Workflow of global proteome sequencing and quantification by mass spectrometry (MS/MS).

Proteomics techniques range from one-dimensional (ID) gel electrophoresis, two-dimensional (2D) gel electrophoresis, Chromatography (liquid and gas) methods to sophisticated mass spectrometry (MALDI-MS, ESI-MS, LC-MS/MS, MALDI-TOF MS, etc.,), which measures mass-to-charge (*m:z* ratio) of ionized peptides to identify proteins (Gupta et al., 2009a). In a typical MS experiment, the proteins are isolated from target cells/tissue/organ/biological fluid, separated by 1D or 2D gel electrophoresis or liquid chromatography, and digested by a sequence-specific protease such as trypsin (Figure 3). The trypsin digests are then purified by affinity chromatography or biochemical fractionation and ionized by electronspray ion (ESI), matrix-assisted laser desorption ionization (MALDI), or surface-enhanced laser desorption ionization (SELDI) before being pushed into the mass spectrometer to measure the *m:z* ratio. The *m:z* ratio can be measured in quadrupole (Q), ion trap (e.g., quadrupole ion trap or QIT and linear ion trap or LIT), time-of-flight (TOF), quadrupole mass filters (QMF), ion cyclotron resonance (ICR), high-resolution orbitraps, or a hybrid of mass spectrometers. The MS spectra are then matched with protein databases to identify proteins using a variety of algorithms that usually come in-built with the MS machines (e.g., SpectraMill^TM^) (Gupta et al., 2009b). Several methods have also been developed for relative or absolute quantification (AQUA) of proteins and identification of post-translational modifications by modified MS such as selected reaction monitoring (SRM), isotope labeling of amino acids in cell culture (SILAC), isotope-coded affinity tags (ICAT), isobaric mass tagging (iTRAQ), etc.

The number of proteomics studies associated with reproductive problems has been increased dramatically in the last decade (Peddinti et al., 2011). The research applications in proteomics range from early growth and development to postmortem events important for meat quality (Yarmush and Jayaraman, 2002; Bendixen, 2005). One of the major areas of interest in proteomics is finding out robust protein biomarkers that could be useful in disease surveillance, monitoring the health and wellbeing of animals, elucidating disease mechanisms, and assessing pharmacologic response to therapeutics (Oskoueian et al., 2016) (Muhanguzi et al., 2022). Proteomics can also be applied for different animal products post-harvest like meat, milk, cheese, etc., to identify genetic variants with desirable traits for selection and breeding (Almeida et al., 2015).

Proteomics has also enabled the identification of candidate protein markers of fertility for molecular breeding. The LC-MS/MS analysis of pig sperm revealed eight fertility-related proteins over-represented in Tibetan pigs having heritable adaptation to hypoxic environments (Zhao et al., 2021). In another study, analysis of seminal plasma proteins by LC-MS/MS found a consistent correlation of 1,343 proteins with fertility (Willforss et al., 2021). Thus, identifying fertility markers by proteomics can help identify fertile bulls to reduce non-return rates (NRR) and increase productivity. Proteomic tools can also be harnessed to identify superior genetic variants to dietary response and muscle growth for selective breeding. By a newly described transcriptome-assisted label-free shotgun proteomics method, Mullins et al. (2021) identified 24 differentially abundant proteins in liver tissues from cattle that were fed *ad libitum* or restricted diet. Identifying protein markers by proteomics could help the selection of genetic variants for compensatory growth upon undernutrition, which may accelerate genetic gain and increase profitability (Mullins et al., 2021).

## Metabolomics

**FIGURE 4:**
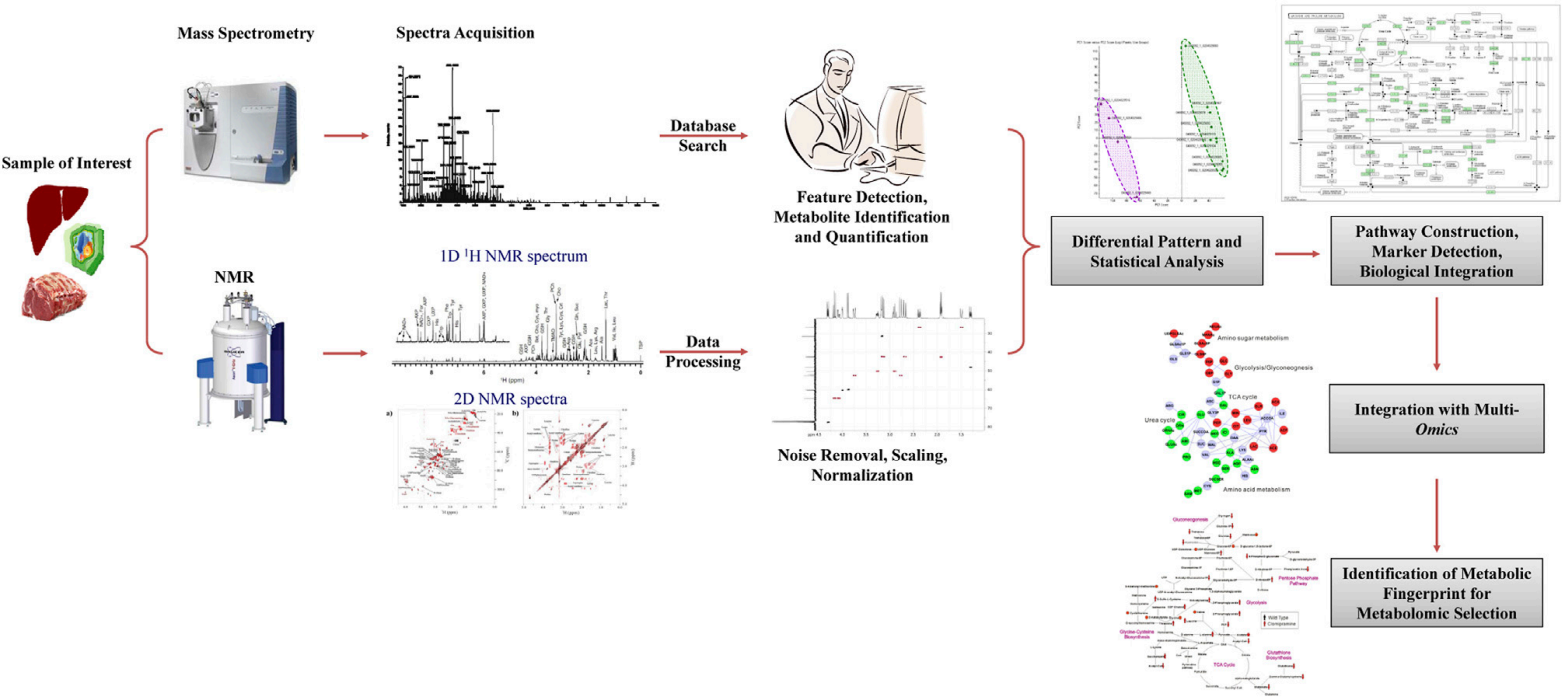
Workflow for application of metabolomics on genetic selection of animals.

An emerging area in the application of *omics* tools is the interrogation of the metabolome. Metabolomics is a comprehensive, qualitative and quantitative study of all the small molecules in an organism (Kalaiselvi et al., 2019; (Lippa et al., 2022). Metabolomic tools are being increasingly used to generate an unbiased global profile of metabolites in samples (i.e., untargeted analysis) or to quantify with high sensitivity a small panel of metabolites (targeted analysis) (Riggs et al., 2017; Evans et al., 2020). In dairy cattle, many potential biomarkers of milk yield and quality have been detected by studying the metabolome of different body fluids (Sun et al., 2015). One advantage of profiling metabolites is exploring the impact of metabolism on systemic health by monitoring the production and further metabolism of compounds present in the diet, digesta, and plasma (Seidel et al., 2014). It can also be used to evaluate feed conversion efficiency, metabolic response of animals to environmental conditions, and estimation of production efficiency and carcass quality traits (Jorge-Smeding et al., 2021; Martin et al., 2021; Artegoitia et al., 2022). Studies have also shown that the heritability of water-soluble compounds such as free amino acids, nucleotides, and sugars in beef was less than 0.30 and varied with animal age (Sakuma et al., 2016). However, these water-soluble compounds were negatively correlated with carcass weight and beef marbling standard at the genetic level. Thus, metabolomics may help identify animals with high carcass quality for breeding. In another study, metabolic profiling of muscle by GC-MS and LC-MS could distinguish grass- and grain-fed cattle with 100% predictive accuracy (Carrillo et al., 2016). These results suggest that metabolic signatures could be a good indicator of animals’ feeding habits and carcass quality and, therefore, could be utilized to select animals with desired traits (Figure 4).

Metabolomic selection is an emerging breeding technology based on nuclear magnetic resonance (NMR) or LC-MS metabolomics (Evans et al., 2020; Lippa et al., 2022). The NMR spectra of biological samples can be analyzed for chemical shifts, peak intensities, and coupling patterns to identify and quantify various metabolites and generate NMR fingerprints of the sample (Figure 4). Metabolomic studies on muscle and fat from cattle, pigs, and poultry have shown tissue- and species-specific differences in metabolites with specific compounds detected in each species (Ueda et al., 2018). The GC-MS could also distinguish between cattle breeds (Ueda et al., 2018). Thus, comparing NMR spectra from different animals such as those from low- and high-performing animals may help identify NMR fingerprints in high-performing animals. Such NMR fingerprints can then be used for the genetic selection of animals for breeding purposes (Figure 4). Metabolomic analysis of muscle from Nellore cattle having high- or low-growth traits revealed that high growth animals had a distinct metabolic profile with a higher concentration of specific metabolites affecting protein and fatty acid metabolism (Cansolo et al., 2020) that can be harnessed for selection of animals for growth.

High-resolution MS (HRMS) can detect metabolites at nano-to the pico-molar concentration of metabolome and, therefore, can provide a better landscape of metabolites than NMR (Goldansaz et al., 2017). The MS is usually combined with separation techniques such as capillary, liquid, or gas chromatography, depending on the polarity and lipophilicity of the metabolites of interest. The separated molecules are ionized by ESI, electron ionization (EI), chemical ionization (CI), or atmospheric pressure CI (APCI) and evaluated for *m:z* ratio in the mass spectrometer based on TOF, Fourier transformation ion cyclotron resonance (FT-ICR) or orbitrap to obtain structural information for identifying the metabolites. Several databases such as METLIN, Human Metabolome Database (HMDB), and MassBank are available that can be used to match the MS spectra of metabolites for their identification. A number of statistical and bioinformatic tools can then be applied to discover molecular pathways involved in the generation of critical metabolites. Software such as MetaboAnalyst and Kyoto Encyclopedia of Genes and Genomes (KEGG) can be used for multivariate analysis and visualization of metabolic pathways. The correlation analysis between animal performance parameters and metabolic profiles may help identify key metabolic markers of animals’ performance for genetic selection. Metabolomics has also been used in GWAS for metabolite-featured phenotyping and animal breeding (Fontansesi, 2016).

The metabolomic tools can also be combined with molecular breeding tools such as WGS and high-density SNP chips to increase the accuracy of genetic selection and livestock breeding (Wang and Kadarmideen 2020) (Ehret et al., 2015). Given that genomic prediction to predict breeding values based on phenotypic, pedigree, and genomic data is insufficient to describe the genetic potential of animals, incorporating the whole-metabolomic data in the genomic prediction equation may play a crucial role in increasing the genetic gain by increasing the accuracy of selection. The latter is further substantiated by the fact that metabolites represent cells’ ultimate physiological response and thereby represent a link between genotype and phenotype (Wang and Kadarmideen 2020). GWAS-based studies, using SNP chips and LC-MS metabolomics, the identified mechanism underlying the genetic variation in pigs for feed efficiency (Banerjee et al., 2020; Wang and Kadarmideen 2020). Integrating high-density SNP data and metabolite information with predictive value was also found to help improve the accuracy of genetic selection in cattle (Ehret et al., 2015). The power of metabolomics is that it non-invasively detects subtle phenotypic changes, innate phenotypic propensities, and dietary responses in livestock research, breeding, and assessment through new varieties of bio-samples such as semen, amniotic fluid, saliva, and urine.

Wu et al. (2021) found that small metabolite profiling of pig feces by LC-MS metabolomics correlated with their feed efficiency and can be used as a reference for selecting animals with high feed conversion efficiency and responsiveness to new feed additives (Wu et al., 2021). Given that fecal metabolome are reflections of intestinal microbiota, cellular metabolism and digestion/absorption of nutrients in the gut (Zierer et al., 2018; Malheiros et al., 2021), the metabolites present in the feces could indicate their feed conversion efficiency (Wu et al., 2021). Fecal metabolome was also shown to change as a function of stress in beef cattle (Valerio et al., 2020). Thus, metabolic profiling of fecal matter may be used to identify animals with “metabolic fingerprints” that are known to exist in animals of high feed conversion efficiency or tolerance to stress. Such animal can then be selected for breeding purposes. Metabolomics has also been used to study the effect of genetic selection on indirect genetic effects (IGE) in breeding programs (Dervishi et al., 2021). Future metabolomics research may be integrated with *multi-omics* experiments using various analytical platforms/techniques (e.g., ICP-MS, MSI, and fluxomics) by using more sensitive platforms, such as ESI-MS, to get more accurate information.

## Metagenomics

Metagenomics is the collection and analysis of genetic material (genomes) from a mixed community of organisms. Metagenomics is an area of considerable research interest, particularly in ruminant animals to study microbial communities in rumen and milk. In metagenomics, genomic sequencing tools are used to identify the complex structure of the rumen microbiota and their changes in response to diet in concert with the host ruminant genome. These rumen microbiotas may influence a range of phenotypes in the host, including feed efficiency, the inflammatory state in the digestive tract, and volume of methane production in the rumen (Morgan et al., 2014; Ritchie et al., 2015). Metagenomics is also the best way to reveal modern species’ phylogenetic and evolutionary relationships with the natives and ancestors of livestock and poultry (Sahu et al., 2017). Other important applications of metagenomics in livestock improvement are to identify the disease-resistant strains for drug of choice and information generation for genotype and environmental interactions for better control over management (Sahu et al., 2017).

**FIGURE 5:**
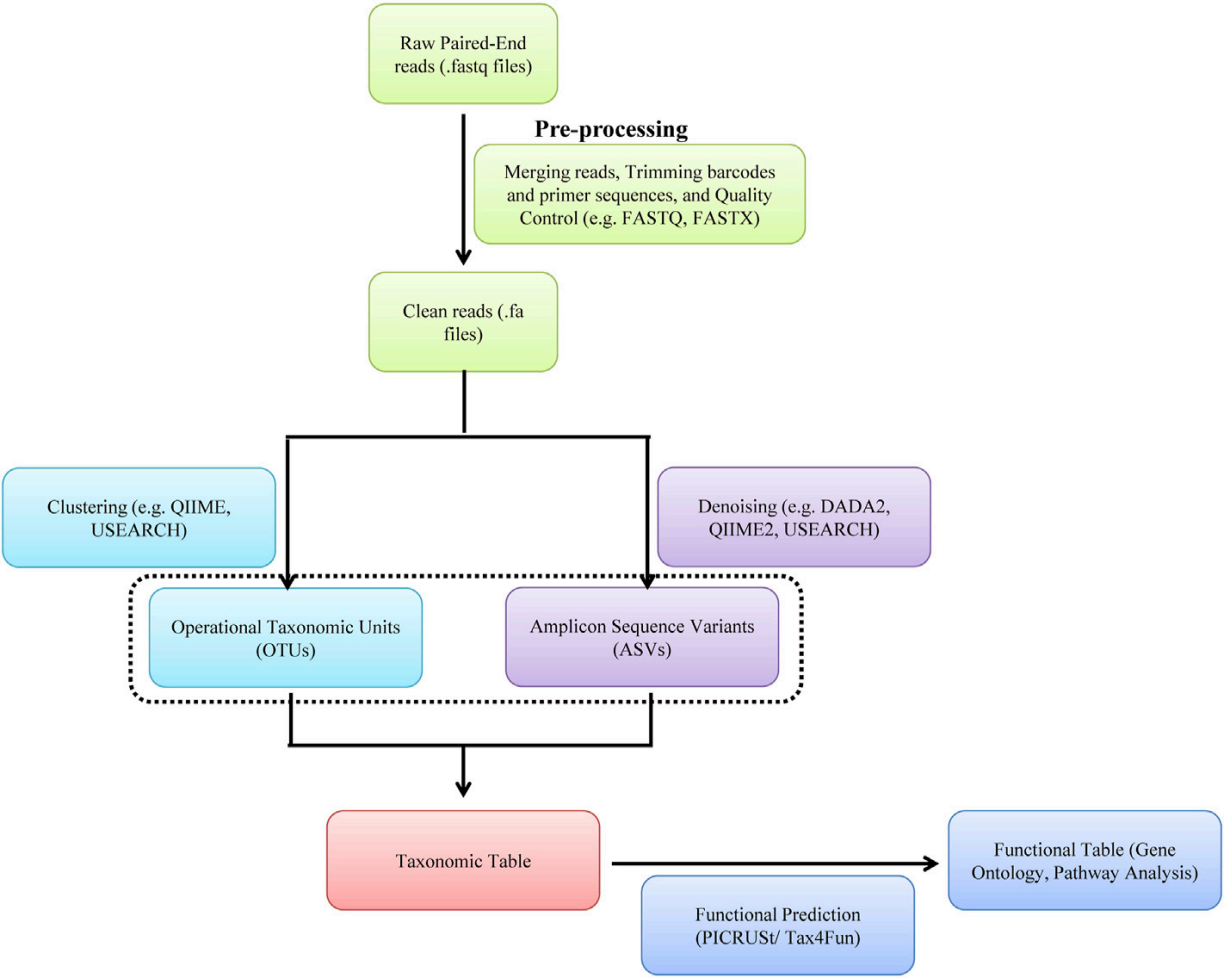
A software pipeline for analysis of amplicon sequencing of bacteria. Each type of experiment has distinct requirements and challenges but there is a common workflow/pipeline.

**FIGURE 6:**
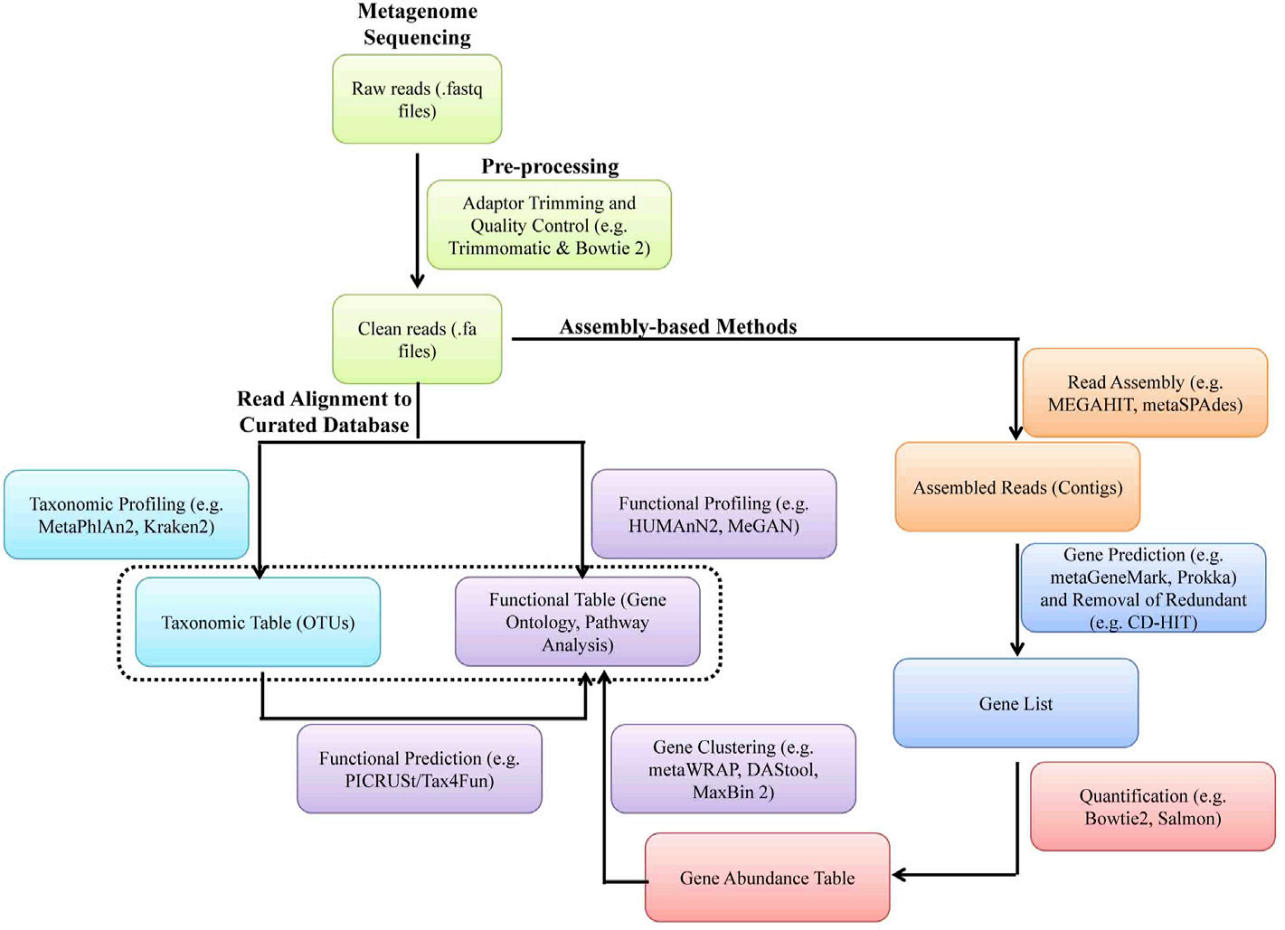
A software pipeline for analysis of whole genome metagenomic sequencing data. Each type of experiment has distinct requirements and challenges but there is a common workflow/pipeline.

A typical metagenomic experiment involves isolation of genomic DNA from microbial population and amplicon sequencing of 16 rRNA hypervariable V3-V4 region of bacteria and/or WGS by NGS (e.g., Illumina^TM^ sequencing) or the third-generation sequencing [e.g., Oxford Nanopore Technology^TM^ (ONT) and PacBio^TM^] (Figures 5, 6). The DNA reads obtained from WGS data are assembled computationally to obtain larger DNA sequences and identify the operational taxonomic units (OUTs) of the microbes. Statistical tools can then be utilized to estimate richness (number of taxonomic groups) and evenness (distribution of abundances of the groups) of various microbial populations by computing alpha and beta-diversities. A number of tools such as Mothur, QIIME2 (Quantitative Insights Into Microbial Ecology), DADA2 (Divisive Amplicon Denoising Algorithm), Usearch etc. are available for amplicon sequencing of 16 rDNA in bacteria. A typical bioinformatics pipeline and relavent tools for analysis of amplicon sequencing is shown in Figure 5. On the other hand, while WGS allows high analysis of entire community of microbs, including viruses and fungi, they are relatively expensive, time consuming and computationally demanding. A bioinformatic pipeline and tools for WGS analysis of WGS is shown in Figure 6. The details of various metagenomic pipelines for amplicon sequencing and WGS can be seen elsewhere (Florian et al., 2019).

Metagenomic studies have been used extensively in cattle, pigs, and horses to understand the importance of the microbiome in the gut and mammary microbiome and their relation to feeding efficiency, immunity, and mastitis (Chen C. et al., 2021; Gomez et al., 2021). Metagenomics has shown that gut microbiota can affect feed intake, feed conversion ratio, and production traits such as daily weight gain and back-fat thickness in pigs (Aliakbari et al., 2021; Jiang et al., 2021; Tiezzi et al., 2021). It can also relieve immune stress and help maintain homeostasis in the intestine (Sun et al., 2021). Similarly, parasitic infestations of tapeworm in horses were also found to alter the colonic microbiome (Slater et al., 2021). Such changes in gut microbiota showed implications in individual animals’ performance and metabolic health. An improved understanding of gut microbiota by metagenomics can, therefore, help to genetic selection of animals for better animal health and productivity. The meganomic profile of fecal matter or oral swab may be used to identify animals with “microbial fingerprints” that are known to exist in animals of high genetic merit and can subsequently be used for breeding purposes. Thus, metagenomics may offer a non-invasive means of animal selection and breeding. Metagenomic studies have also revealed that the milk microbiome varies with health status (e.g., mastitis, endometritis, bacteremia, etc.,), age, parity, lactation duration, and feed composition (Bach et al., 2021). Consequently, characterization of milk microbiomes may help identify novel “microbial fingerprints” for healthy mammary glands and genetic selection of healthy dairy animals (Andrews et al., 2019).

## Epigenomics

Epigenomics is another branch of *omics* technology that deals with studying epigenetic changes in a cell. Epigenetic changes regulate gene expression without changing the actual DNA sequence. Epigenetic modifications can be altered by external or internal environmental factors such as diet, exercise, drugs, and chemicals and can change gene expression and define specific phenotypes. Mapping epigenomics components in many cell types helped identify millions of putative regulatory elements (Zentner and Henikoff, 2015). A whole-genomic bisulfate sequencing identified breed-specific hypomethylated regions that were associated with male fertility (Chen S. et al., 2021). Epigenomic biomarkers of male fertility were also identified in the genome-wide DNA methylation map of pig testis (Wang and Kadarmideen 2019). However, studies on global-level epigenomics for genetic selection and breeding of animals are very limited.

## Analysis of Omics Data

*Omics* technologies generate a voluminous amount of complex data in gigabyte to terabyte range (hence, called Big Data) that are difficult to handle by traditional data management tools (Angerer et al., 2017). Expertise from different biological fields, skilled and knowledgeable bioinformaticians, statisticians, and computer scientists are required to analyze and interpret these data (Riggs et al., 2017). The tremendous high-dimensional data resulting from a large number of experimental variables (e.g., physiological state, age, sex, parity, nutritional status, experimental design, etc.) and simultaneous evaluation of multiple genes/proteins/metabolites/transcripts, etc. requires implementation of complex statistical techniques and models to avoid spurious results and misinterpretation of research data. Various open-source and commercial bioinformatics softwares are now available in online and offline modes in R packages of Bioconductor, EMBOSS, Galaxy, Staden, Biophython, Bioconda, Linux, etc., for various *omics* data analysis (Table 2).

**TABLE 2:** Overview of some free bioinformatics software for integrating information across several *omics* techniques.

Name	Integration of types of omics	References and URL
Cytoscape	Mainly protein-protein, protein–DNA, and DNA–DNA interactions, but plug-ins (apps) are available for all types of omics	https://cytoscape.org/; Shannon et al. (2003)
MOFA	All types (multi-omics)	https://github.com/bioFAM/MOFA; Argelaguet et al. (2018)
LUCID	Mainly genomics and metabolomics; integration of phenotypic data	https://github.com/USCbiostats/LUCIDus; Peng et al. (2020)
MultiDataSet	Epigenomics, transcriptomics, assay data, feature data, phenotypic data stored in a single object	https://bioconductor.org/packages/release/bioc/html/MultiDataSet.html; Hernandez-Ferrer et al. (2017)
Logicome Profiler	Applied to genomics and metagenomics, but applicable to any omics data	https://github.com/fukunagatsu/LogicomeProfiler; Fukunaga and Iwasaki, (2020)
CoCoNet	Integration of GWAS and gene expression data	http://www.xzlab.org/software.html; Shang et al. (2020)
NEO	Integration of GWAS and gene expression data	https://horvath.genetics.ucla.edu/html/aten/NEO/; Aten et al. (2008)
WGCNA	Mainly gene-expression data, but can be applied to other omics	https://horvath.genetics.ucla.edu/html/CoexpressionNetwork/Rpackages/WGCNA Zhang and Horvath, (2005); Langfelder and Horvath, (2008)
DIABLO in mixOmics	All types (multi-omics)	http://mixomics.org/mixdiablo Tenenhaus et al., (2014); Rohart et al., (2017); Singh et al. (2019)

The Cancer Genome Atlas (TCGA)	RNA-Seq, DNA-Seq, miRNA-Seq, SNV, CNV, DNA methylation, and RPPA	https://cancergenome.nih.gov/
Omics Discovery Index	Genomics, transcriptomics, proteomics, and metabolomics	https://www.omicsdi.org/ Perez-Riverol et al. (2017)
OMICtools	NGS, microarray, polymerase chain reaction (PCR), MS and NMR technologies	http://omictools.com/
NGOMICS-WF	Metagenomic, metatranscriptomic, RNA-seq and 16S data	https://github.com/weizhongli/ngomicswf
Paintomics	Integrated visual analysis of transcriptomics and metabolomics data	http://www.paintomics.org
GalaxyP, GalaxyM	Integrated omics analysis, proteomics informed by transcriptomics analysis	https://usegalaxy.org/
Omics Integrator	Integrate proteomic data, gene expression data and/or epigenetic data using a protein-protein interaction network	http://fraenkel.mit.edu/omicsintegrator, https://github.com/fraenkel-lab/OmicsIntegrator
IMPaLA	Joint pathway analysis of transcriptomics or proteomics and metabolomics data	http://impala.molgen.mpg.de

Data handling is a vital component of analyzing raw data from *omics* experiments for their correct biological interpretation. Data handling must address issues related to data filtering, imputation, transformation, normalization, quality control, and scaling (Li et al., 2022). Several algorithms and pipelines are now available for the analysis of various *omics* data, including transcriptomics (Figure 3), proteomics (Figure 4), and metagenomics (Figure 4). However, using one pipeline or tool may yield different results from other pipelines. One approach to avoid this problem is to use multiple well-documented analysis pipelines for each step in the pipeline (Misra et al., 2019). The detailed discussion on various *omics* and *multi-omics* pipelines is beyond the manuscript’s scope. There are excellent reviews available on transcriptomic (Wadapurkar et al., 2021), proteomic (Halder et al., 2021), metagenomic (Yang et al., 2021), and metabolomic (Du et al., 2022) pipelines and their integration for *multi-omics* (Subramanian et al., 2020; Reel et al., 2021), which can be referred. GitHub (https://github.com/danielecook/Awesome-Bioinformatics) and Biostars (https://www.biostars.org/) are also good sources of various updates on *omics-*related softwares and data analysis, respectively.

## Challenges in Applications of Omics Strategies in Livestock Selection and Breeding

Applications of omics technology to explore the full potential of livestock face many practical challenges. Some of those challenges are as follows:

### Proper Maintenance of Data

Data recording and handling of raw data is a big challenge for breeders. To avoid errors and bias in data processing and analysis, suitable cutoffs (e.g., microbial relative abundance, gene expression threshold, metabolite similarity, differential expression cutoff, enriched function cutoff, significant impact value of pathways), data preprocessing options (e.g., data baseline filtering and calibration, peak alignment, deconvolution analysis, peak identification), data normalization, data transformation, and data scaling methods should be carefully considered and addressed within each study. Database construction is an important way for data storage and data maintenance. One such database is ASlive, which has been designed for livestock to capture alternative splicing events in heterogeneous samples from a wide range of tissues, cell types, and biological conditions (Liu et al., 2020). More such databases will accelerate the study and applications of *omics* technology for animal improvement.

### Lack of Phenomics Data

Most organized animal farms maintain the performance records of various economic traits, including production traits, reproduction traits, and growth traits. However, organism-wide phenotypic data of animals during different growth phases, various physiological or production stages, in response to dietary changes or upon their selective breeding (i.e., phenome-level data) are challenging to maintain and are generally missing (Pérez-Enciso and Steibel 2021). Accurate phenomics data on adaptability, fitness, body conformation, disease resistance/susceptibility, production performance, reproduction, and growth characteristics will help better estimate accurate BV and selection of genetically superior animals (Juárez et al., 2021; Pérez-Enciso and Steibel 2021). Particular emphasis should be given for *multi-omics* with other “big data”; for example, those detected by advanced management technologies (e.g., using remote sensors communicating with the Internet of Things to measure physiological and behavioral data, which can be applied to monitor estrus, lameness, or rumination) to have complete data set (Sun et al., 2019). The systematic collection of large data sets from different biological layers will help generate a more holistic understanding of the biological factors affecting the performances of the animals.

### Expertise

*Omics* technology generates enormous amounts of data at the genome, transcriptome, proteome, or metabolome levels. Proper handling of *omics* data and advanced knowledge of statistics and bioinformatics are prerequisites for the adequate utilization of *omics* technology. One should have good knowledge in the above-mentioned fields and computer knowledge to interpret the data generated through *omics* technology. Lack of good expertise may mislead for selection of suitable animals. There are many challenges associated with proper data recording, processing, quality control, normalization, and genetic prediction (Yamada et al., 2021). Breeders, biological scientists, veterinarians, statisticians, and computer scientists should be trained to overcome these problems. All should collaborate to interpret and adequately utilize *omics* data, including phenomics data for animal improvement.

## Conclusion

The goal of animal production is to achieve increased productivity to fulfill human demand while enhancing the health and wellbeing of animals. Population growth, climate change, resource depletion, human health and nutrition, and sustainability are all issues with which the world is grappling. Different new breeding technologies and molecular technologies such as genomic selection, WGS, and gene editing contribute tremendously to the selection and breeding of livestock species for sustainable improvement in productivity and profitability. *Omics* technologies such as genomics, proteomics, transcriptomics, metagenomics, and metabolomics offer powerful analytical tools that can be combined with molecular breeding for the accurate selection of animals for improved productivity. Genomics, in particular, can make conventional breeding and advanced breeding techniques more efficient and precisely targeted by increasing consistency and predictability. Integration of multi-layers of *omics* technology, including phenomics, into the breeding models, will be helpful for proper selection and breeding for animal improvement in the near future.
